# Key Factors in the Complex and Coordinated Network of Skin Keratinization: Their Significance and Involvement in Common Skin Conditions

**DOI:** 10.3390/ijms25010236

**Published:** 2023-12-23

**Authors:** Nives Pondeljak, Liborija Lugović-Mihić, Lucija Tomić, Ena Parać, Lovre Pedić, Elvira Lazić-Mosler

**Affiliations:** 1 Department of Dermatology and Venereology, General Hospital, 44000 Sisak, Croatia; nives.pondeljak@gmail.com (N.P.); lucijatomic2@gmail.com (L.T.); elvira.lazic@gmail.com (E.L.-M.); 2 School of Dental Medicine, University of Zagreb, 10000 Zagreb, Croatia; lovrepedic@gmail.com; 3 Department of Dermatovenereology, Sestre milosrdnice University Hospital Center, 10000 Zagreb, Croatia; enaparac7@gmail.com; 4 School of Medicine, Catholic University of Croatia, 10000 Zagreb, Croatia

**Keywords:** keratinization process, the epidermis, skin desquamation, corneocytes, calcium gradient, keratin expression, desmosomes, tight junctions, gap junctions, aquaporins

## Abstract

The epidermis serves many vital roles, including protecting the body from external influences and healing eventual injuries. It is maintained by an incredibly complex and perfectly coordinated keratinization process. In this process, desquamation is essential for the differentiation of epidermal basal progenitor cells into enucleated corneocytes, which subsequently desquamate through programmed death. Numerous factors control keratinocyte differentiation: epidermal growth factor, transforming growth factor-α, keratinocyte growth factor, interleukins IL-1-β and IL-6, elevated vitamin A levels, and changes in Ca^2+^ concentration. The backbone of the keratinocyte transformation process from mitotically active basal cells into fully differentiated, enucleated corneocytes is the expression of specific proteins and the creation of a Ca^2+^ and pH gradient at precise locations within the epidermis. Skin keratinization disorders (histologically characterized predominantly by dyskeratosis, parakeratosis, and hyperkeratosis) may be categorized into three groups: defects in the α-helical rod pattern, defects outside the α-helical rod domain, and disorders of keratin-associated proteins. Understanding the process of keratinization is essential for the pathogenesis of many dermatological diseases because improper desquamation and epidermopoiesis/keratinization (due to genetic mutations of factors or due to immune pathological processes) can lead to various conditions (ichthyoses, palmoplantar keratodermas, psoriasis, pityriasis rubra pilaris, epidermolytic hyperkeratosis, and others).

## 1. Introduction

The roles of the epidermis are multiple; above all, the protection of the organism from external influences throughout its entire life cycle and, in the case of injury, the role of healing as quickly and efficiently as possible and restoring the continuous barrier. The epidermis is maintained with an incredibly complex and perfectly coordinated keratinization process.

The process of full regeneration of the skin includes keratinocyte proliferation, early differentiation, terminal differentiation, and desquamation, which is important for maintaining healthy skin. This process is maintained by stem cells that self-renew and generate the interfollicular epidermis, hair follicles, and sebaceous glands [1]. The role of the epidermis is to provide an impenetrable barrier, and at the same time it changes every two weeks through a highly regulated proliferation/differentiation process. When normal skin proliferation and differentiation are impaired, skin disease can occur. Skin diseases can be divided into (a) those that have abnormal and premature keratinization occurring within cells or cell groups below the stratum granulosum (e.g., Darier’s disease); (b) those where the keratinization process is both accelerated and incomplete, resulting in the retention of residual nuclei within the stratum corneum (e.g., psoriasis); and (c) those with excessive accumulation of keratin and thickening of the cornified layer or disorders related to the proper detachment of the stratum corneum (e.g., palmoplantar keratodermas, ichthyoses) [2,3]. According to one prominent book in the literature, the group “Disorders of Keratinization” comprises the spectrum of diseases characterized by incorrect epidermal differentiation, such as ichthyosis, palmoplantar keratoderma, erythrokeratodermia, follicular hyperkeratoses, acantholytic dyskeratoses, porokeratoses, and others [4]. Also mentioned in this group are scaly skin diseases such as lichen planus, lichen sclerosus, ichthyosis, pityriasis rubra pilaris, Darier disease, and keratosis pilaris, as well as other skin diseases.

Considering the complexity of this topic, understanding the molecular mechanisms involved in proper keratinocyte proliferation and differentiation is essential for uncovering the complete pathogenesis of various skin disorders and providing potential targets for therapeutic interventions.

## 2. Basic Features of the Keratinization Process

Keratinization (cornification) is a process by which keratinocytes differentiate, moving from the basal layer to the distinct outer layer called the stratum corneum, where they become specialized cells known as corneocytes. These cells are devoid of a nucleus and contain keratin filaments bound by a filament-aggregating protein filaggrin that contributes to the resilience and durability of the stratum corneum. It is a keratin filament-aggregating protein that serves as a major structural component of the stratum corneum that allows the skin to withstand different mechanical and chemical insults [5,6,7]. This arrangement contributes to the resilience and durability of the stratum corneum and allows the skin to withstand different mechanical and chemical insults. Moreover, the differentiation process of keratinocytes allows the epidermis to be a perpetually regenerating tissue. Disruptions in the keratinization process may lead to various skin disorders [8]. As a result, a feature commonly mentioned in various dermatological diseases is hyperkeratosis, characterized by an increased thickness of the stratum corneum.

Associated with the keratinization process is the physiological process of skin desquamation, which is crucial for maintaining healthy skin. The epidermis consists of layers progressing towards the surface: stratum basale, spinosum, granulosum, lucidum (only in the palmoplantar epidermis), and corneum. The process of desquamation essentially involves the differentiation of basal progenitor cells in the epidermis into enucleated corneocytes, which desquamate through programmed cell death, though differently from the process of apoptosis [7]. Although apoptosis and keratinization share similarities, such as the loss of the nucleus and other cell organelles, the residual product of epidermal keratinocytes, known as corneocytes, is not removed by phagocytosis but instead maintains barrier function until desquamation [9]. Therefore, the entire epidermis from the stratum basale to the stratum corneum changes within 14 days [10]. This process does not occur simultaneously and uniformly in each place, but there is a daily, asymmetrical “shedding off” of the upper layer of the stratum corneum, while basal keratinocytes continuously undergo division [9]. In the keratinization process, as keratinocytes transition from stratum granulosum to corneum, their plasma membrane is transformed into a keratinized envelope of corneocytes. This is achieved by covalently crosslinking envoplakin, periplakin, involucrine, loricrine, and filaggrin proteins into the plasma membrane, which is then coated with non-polar hydrophobic lipids [11].

Keratinocyte proliferation and desquamation are critical processes in developing and maintaining healthy skin [11]. In the skin’s normal cycle, keratinocytes are gradually produced in the epidermal basal layer, then migrate through several layers, and eventually shed off at the surface. Any dysregulation of these processes can give rise to various skin conditions. Aberrations in keratinocyte growth and turnover, for example, can lead to proliferative and retention hyperkeratosis and related disorders. Keratinocyte proliferation disorders are skin disorders characterized by excessive and rapid, novel cell production. Conditions such as psoriasis, pityriasis rubra pilaris, and epidermolytic hyperkeratosis belong to this group, psoriasis being the most common among them. Psoriasis, a chronic autoimmune disorder, results from an aberrant reactivity and signaling between innate immune cells, adaptive immune cells, and resident skin cells. Such crosstalk leads to a seven-fold increase in the number of cycling epidermal cells, clinically evident as erythematous plaques covered by silvery scales. On the contrary, when the skin cells are produced at a normal rate but shedding is disrupted, retention disorders occur. Some examples of keratinocyte retention include keratosis pilaris and ichthyoses, a group of diseases with underlying genetic mutations manifested in persistent dry and scaly skin [12,13].

Over the course of a lifetime, the epidermis must continually provide an impenetrable barrier while at the same time changing rapidly, every two weeks, and retaining the ability to heal wounds. The highly regulated proliferation/differentiation program of the epidermis fulfills these different roles. The ability of the epidermis to constantly renew itself and heal wounds lies in the fact that it contains stem cells, which are progenitor cells that can self-renew and create differentiated progeny over a long period of time. The tissue microenvironment in which stem cells normally exist is called their niche. Niches contain direct cell-cell and cell-matrix communication, soluble secreted factors, nervous factors, and local tissue mechanical properties. Different studies have shown that niche stem cells are scattered throughout the basal layer or at the tops or at the bottom of rete ridges [14]. Notwithstanding their position, progenitor cells in the basal layer mostly produce differentiated progeny that form in the columns immediately above them [15].

The progeny of epidermal stem cells undergo terminal differentiation to generate the interfollicular epidermis, hair follicles, and sebaceous glands [1]. It appears that distinct populations of stem cells exist within these structures, and the differentiation pathway chosen by their progeny is largely determined by local signals from the microenvironment [16]. Understanding the factors that influence the behavior of stem cells is essential for the advancement of the field of regenerative medicine [17]. The key components of the niche are the following: homo- and heterotypic cell-cell contact, cell-extracellular matrix, adhesion, mechanical stimuli, secreted factors, and physiological factors such as oxygen and pH [18]. The Notch pathway plays an important role in the regulation of epidermal differentiation. The expression of Notch ligands, effectors, and receptors in embryonic and adult skin occurs in an intricate and dynamic pattern. Experimental studies involving genetic ablation or activation of the pathway have unveiled its promotion of differentiation in the hair follicle, sebaceous gland, and interfollicular epidermal lineages. Additionally, Notch acts as an epidermal tumor suppressor. The interplay of Notch signaling with various pathways is crucial for these functions, acting through both retinal pigment epithelium (RBP)-Jκ-dependent and independent mechanisms. The impact on differentiation is observed at both the autonomous and non-autonomous cell levels, with Notch influencing stem cell clustering by modulating cell adhesion [19].

Mitotic activity of basal progenitor cells is of the most importance for normal epidermal turnover and wound healing. Basal progenitor cells are labeled by the expression of keratins 5 and 14 [20]. Basal keratinocytes create spinous cells during epidermal homeostasis. Cell production in the suprabasal cell layers is driven by mitotic spindle reorientation and asymmetric cell divisions during embryonic development [3,21]. Differentiation from basal to spinous cells is a highly regulated transition. Spinous cells switch from a mitotic keratin 5/14 expression to a postmitotic keratin 1/10 expression. Furthermore, these cells have increased regulation of desmosomes, which gives them a spiny appearance in histological sections. Differentiation also changes the composition of desmosomes where desmoglein (Dsg) levels (Dsg3) decrease through differentiation, while Dsg1 is upregulated [22].

The identifying feature of granular cells are keratohyaline granules. These granules consist of keratin, profilaggrin, and loricrin. After secretion, keratins, loricrins, as well as other proteins such as prolines, become highly cross-linked by transglutaminase to the plasma membrane and form cornified envelopes. Profilaggrin is eventually metabolized into amino acids to provide hydration and serve a UV protective function. The role of granular cells is to prevent fluid loss, using tight junctions, which are intercellular adhesions that limit the flow of fluids and ions and act as a barrier to membrane diffusion. The last function of granule cells is programmed cell death. The resulting cornified envelopes are not living cells, so the nuclear and cytoplasmic contents must be eliminated.

Stratum corneum consists of cornified envelopes, which are products of terminal differentiation in the epidermis. Cornified envelopes are acellular and anuclear structures. Their core consists of keratins surrounded by a highly crosslinked network of proteins [14]. Structural changes accompanying differentiation are highly regulated by a cascade of pathways, including signaling pathways, epigenetic factors, transcriptional control, and posttranscriptional regulation. Notch signaling is a factor responsible for the transition of basal cells to spinous cells [1]. While Notch signaling is inactive in basal cells, it is activated in spinous cells. Inhibition of Notch signaling prevents many aspects of spinous cell fate, while its activation in basal cells is sufficient for some aspects of spinous differentiation. Grainyhead-like (GRHL) transcription factors are also key promoters of epidermal differentiation. Most notably, GRHL3 is required for efficient barrier formation, in part through regulation of transglutaminase-1 expression [23]. Corneodesmosomes are the main intercellular adhesive structures in the stratum corneum [24]. Corneodesmosin is secreted by lamellar bodies into the extracellular spaces surrounding stratum granulosum and its cells (SG cells) and then integrated into desmosomes, resulting in the formation of corneodesmosomes [7]. The crucial role of corneodesmosomes is exemplified by the finding that the lack of a corneodesmosome component, specifically corneodesmosin, leads to premature detachment of the stratum corneum in both mice and humans [25]. Considering the above, it is clear that corneodesmosomes play a vital role in maintaining the cohesion of the stratum corneum, making them a key component of the functional protective barrier of human skin [24].

However, numerous factors control keratinocyte differentiation, including epidermal growth factor (EGF), transforming growth factor-α (TGF-α) and transforming growth factor-β (TGF-β), keratinocyte growth factor (KGF), as well as IL-1-β, IL-6, and elevated vitamin A levels. So, EGF and TGF-α, via the EGF receptor, which is located in basal cells, promote a mitogenic effect on basal cells [11]. Notably, KGF is a member of the fibroblast growth factor (FGF) family of mitogens. While other FGFs act on different cells in proliferation and differentiation, KGF appears to specifically influence epithelial cells. KGF acts predominantly in a paracrine manner, providing a robust stimulus for the proliferation and migration of epithelial cells [26]. KGF production is stimulated by IL-1-β and IL-6; thus IL-1-β and IL-6 increase keratinocyte proliferation. Furthermore, elevated vitamin A levels lead to mucous metaplasia in normally cornified epithelia, wherein cornified surface cells are substituted by noncornified cells from the lining of the mucosa. Conversely, a deficiency in vitamin A can result in the opposite effect, namely squamous metaplasia [11]. Finally, TGF-β inhibits DNA synthesis in basal cells while promoting terminal differentiation. Both basal and suprabasal cells secrete TGF-β [11].

Understanding the basic features of the keratinization process is essential for unraveling the pathogenesis of various skin disorders and may provide potential targets for therapeutic interventions.

## 3. Structure of the Epidermis and Keratinocyte Features Important for Normal Epidermal Structure

The stratum basale in the interfollicular epidermis contains cylindrical keratinocytes along with clusters of stem/progenitor cells capable of asymmetric division. Progenitor cells divide and produce both an identical transit-amplifying stem cell and a cell that begins differentiating into keratinocytes after several divisions, thus maintaining the continuous ability to divide. Keratinocytes that move into terminal differentiation separate from the basement membrane and move suprabasally due to the inactivation of integrins and extracellular matrix receptors that bind them to the basement membrane. These differentiating keratinocytes undergo a keratinization process (through the stratum spinosum, granulosum, and corneum) in the projection of the column distal to the initial stem cell. Above the layer of basal cells there are polyhedral cells of the stratum spinosum whose protein synthesis activity increases, indicating the beginning of a biochemical change in keratinization [11].

The cells of the next layer, the stratum granulosum are flatter and broader and contain basophilic granules of keratohyaline and lamellar bodies (Odlanda) [11]. The stratum granulosum consists of at least three layers of flattened granular cells (listed from the stratum corneum to the stratum spinosum termed SG1, SG2, and SG3) [27,28,29,30]. It is important to note that tight junctions seal the intercellular spaces in the SG2 layer and divide the extracellular space into two compartments. SG1 cells above the narrow junction line are ready for the final keratinization process and the transition into corneocytes in the stratum corneum [28,30].

Lamellar bodies are produced within the Golgi complex and stored as intracellular vesicles within the cytoplasm of SG3 cells. The contents of lamellar bodies (polar lipids, glycosphingolipids, free sterols, and phospholipids) are essentially exocytosed from the apical surface of SG2 layer cells; they move towards the space above the narrow junction line and form a lipid layer that coats corneocytes. After the formation of the lamellar body in the Golgi complex, the uptake of polar lipids into the lamellar body is regulated by ATP-binding cassette transporter 12 channels (ABCA12) located at the boundary membrane of the body. Through exocytosis of lamellar body contents, polar lipids are enzymatically converted into nonpolar ones (glycosphingolipids are hydrolyzed into ceramides, and phospholipids are converted into free fatty acids). The lamellar bodies are also rich in acylceramides, and the fusion of the lamellar body with the keratinocyte plasma membrane gradually increases the acylceramide content in the plasma membrane’s lipid bilayer [5]. The acylceramides then covalently bind to the protein coat, which is created simultaneously from within the cell by transglutaminase 1, eventually replacing the plasma membrane entirely [31,32]. Acylceramide is an unusual ceramide whose N-acyl chain is composed of an omega-hydroxylated ultra-long chain of fatty acids. The fatty acid chain of the ultra-long acylceramide chain is thought to extend throughout the entire lipid layer that coats the corneocytes, effectively bridging and connecting the two membranes of adjacent corneocytes [33]. Lamellar bodies may also contain hydrolytic enzymes to modify lipids, corneodesmosins to modify corneodesmosomes, antimicrobial peptides, proteases, and protease inhibitors crucial for regular desquamation [5].

The protein content of keratohyaline granules includes loricrin and profilaggrin [14]. These proteins are secreted under the signal of increased intracellular Ca^2+^ in keratinocytes of the granular layer. Simultaneously, envoplakin, periplakin, and involucrin, located within the cell, covalently bind to the inner surface of the plasma membrane, and the binding process is carried out by transglutaminase 1 (also dependent on calcium concentration). At a later stage of cornification, the protein coat formed by involucrin, envoplakin, and periplakin on the keratinocyte surface is further reinforced through a covalent cross-link with loricrin. Loricrin is a hydrophobic protein that plays a role in waterproofing the cornea, ultimately becoming its main component at the end of the keratinization process. Profilaggrin is also stored within the keratohyaline granules [14]. In the keratinization process, profilaggrin matures into filaggrin monomers, which subsequently bind to keratin intermediate filaments that are associated with desmosomes. Additionally, they covalently cross-link with each other facilitated by the envoplakin-periplakin-involucrine coatings on the inside of the cell membrane [5,34]. As is generally known, cells possess a cytoskeleton made of three types of protein fibers: microfilaments, intermediate filaments, and microtubules [5]. Microfilaments are the narrowest components of the cytoskeleton and have a 7 nm diameter, whereas microtubules are the widest filamentous components of the cytoskeleton with a diameter of around 20 nm. Intermediate filaments, as their name implies, have a diameter that falls between that of microfilaments and microtubules. These long, unbranched, and chemically stable filaments act as a structural framework within the cytoskeleton by aggregating into 7 to 12 nm wide bundles. The keratins composing intermediate filaments are exclusively expressed in epithelial cells, irrespective of the germ layer from which these cells originate. Due to a high degree of molecular diversity, keratins are an important category among different families of intermediate filaments [5].

In the stratum corneum, corneocytes are formed, which have lost all their nuclei and other organelles such as ribosomes, mitochondria, and granules [11]. The corneocyte becomes compact and covers a larger area than the basal cell from which it developed and eventually desquamates. The intercellular space of the stratum corneum is filled with a hydrophobic layer of lipids, while below the stratum corneum, keratinocytes are immersed in the hydrophilic intercellular space. Corneocytes adhere to each other through adhesion complexes called corneodesmosomes. These complexes are formed by exocytosis of corneodesmosin, which integrates into the keratinized envelope of corneocytes within desmosomes, thus forming corneodesmosomes. Additionally, the force of adhesion is formed by the lipid layer [35,36]. Corneodesmosomes are broken down in the outer layers of the stratum corneum, and the most distant corneocytes are separated, one by one, from the most superficial layer by the process of desquamation [37]. The major proteases responsible for corneodesmosome degradation are serine proteases belonging to the kallikrein group. Kallikreins 5, 7, and 14 are isolated in the stratum corneum and are also secreted by lamellar bodies. These kallikreins are initially produced as inactive precursors and become activated through proteolytic conversion, either by autoactivation or by transmembrane serine proteases [38,39]. Activated kallikreins are thought to be inhibited by the direct binding of lympho-epithelial Kazal-type-related inhibitors (LEKTI), which are also secreted into the intercellular spaces via lamellar bodies [40]. The activity of LEKTI in the lower stratum corneum is considered key to preventing premature desquamation by inhibiting corneodesmosome degradation [41]. Thus, LEKTI binding to kallikrein depends on pH. It is also important to note that during corneocytes’ movement towards the upper layers of the stratum corneum, filaggrin is degraded by proteases into free amino acids, and their derivatives constitute natural emollient factors [5].

Considering all the aforementioned, it is evident that a number of factors control keratinocyte differentiation. These include EGF and TGF-α, KGF, as well as interleukins IL-1β and IL-6, which can contribute to increased keratinocyte proliferation. Additionally, TGF-β inhibits deoxyribonucleic acid (DNA) synthesis in basal cells and promotes terminal differentiation. Furthermore, high vitamin A levels and changes in Ca^2+^ concentration also influence this process [5].

## 4. The Role of Keratin Expression in the Keratinization Process

Keratins are a multigenic family of proteins—as many as 54 genes encode keratin—that comprise a large proportion of keratinocytes in the epidermis [42]. Keratins can be divided into an acidic group (group I—keratins 9–20) and alkaline group (group II—keratins 1–8) [43]. So, α-keratin is a protein chain made up mostly of the amino acids alanine, leucine, arginine, and cysteine, which form the right-sided α-helix. In the formation of a complex structure, two of these polypeptide chains are further linked by disulfide bonds, using cysteine, to form a left-handed helical structure that is approximately 45 nm long. When formed, these dimers are then aligned with other identical dimers, following the principle of binding the terminal end of one dimer to the terminal of another through disulfide bonds, creating a protofilament. Two protofilaments aggregate into protofibrils and four protofibrils into intermediate filaments. These intermediate filaments can subsequently be condensed into coils of keratin about 7 nm in diameter, either type I, acidic, or type II, alkaline [42].

Cytokeratins 1, 5, 10, 11, 14, and 15 are the primary keratins found in normal epidermal cells, and their distribution in the epidermis varies depending on the stage of differentiation. Hence, cytokeratin 5 is specific to normal basal cells, while cytokeratins 1 and 10 are specific to normal suprabasal cells. Regarding markers that indicate terminally differentiated keratinocytes, loricrin, involucrin, and filaggrin are the proteins used. Furthermore, TGF-β is the key factor governing keratinocyte differentiation, a process that typically lasts around 26 to 28 days, known as the turnover time [44].

Keratins in the skin fundamentally affect the structure and mitotic activity of epithelial cells, thereby maintaining mechanical stress, preserving their structural integrity, ensuring mechanical elasticity, protecting against changes in hydrostatic pressure, and establishing cell polarity [43,45]. They also participate in cell signaling, transport, and differentiation; have the ability to modulate protein synthesis; and participate in wound healing [11,46]. Keratins are markers for keratinocyte differentiation, and research suggests that intermediate filaments directly communicate and regulate several signaling pathways [20,42]. As is known, keratins contribute to the functional integrity and mechanical stability of both individual epithelial cells, as well as the cohesion of epithelial tissues through cell-to-cell contacts [5]. In the 1970s, significant advancements in keratin research took place, among which was the discovery that denatured, soluble keratin proteins could spontaneously self-assemble and polymerize into keratin filaments through in vitro dialysis [47]. Subsequently, different keratin types were found, and the progress in laboratory diagnostics has made identifying and characterizing keratin simpler. The recognition of different types of keratin aids in current diagnostics of various pathological conditions. Since the expression of keratins and some keratin-associated proteins is specific to both the region and the stage of differentiation, they can serve as valuable differentiation markers. Defects in keratin and their proteins may result in disorders of the skin, the oral cavity, or both [5].

Keratins in the epidermis are expressed as a keratin pair of alkaline and acidic keratin. Mitotically active keratinocytes in the basal epidermal layer primarily express the keratin pair keratin 5 and 14, with less abundant expression of keratin 15 (in the absence of keratin 14, keratin 15 can be paired with keratin 5). As keratinocytes move suprabasally toward the stratum spinosum, they withdraw from the cell cycle, reducing the expression of keratin 5 and keratin 14 and inducing differentially specific keratins 1 and 10. Further maturation of keratinocytes into the stratum granulosum results in keratin 2 expression [42]. Some keratins have specific anatomical sites of expression, so keratin 9 is particularly expressed in suprabasal cells of the palmoplantar skin. Keratins 6, 16, and 17 are not solely expressed in the palmoplantar epidermis but also in the keratinocytes of the nail plate, hair follicles, and sebaceous and sweat glands. In addition, this group of keratins is rapidly induced by physical injury and ultraviolet radiation, as well as in hyperproliferative disorders [48,49,50,51].

**Figure 1:**
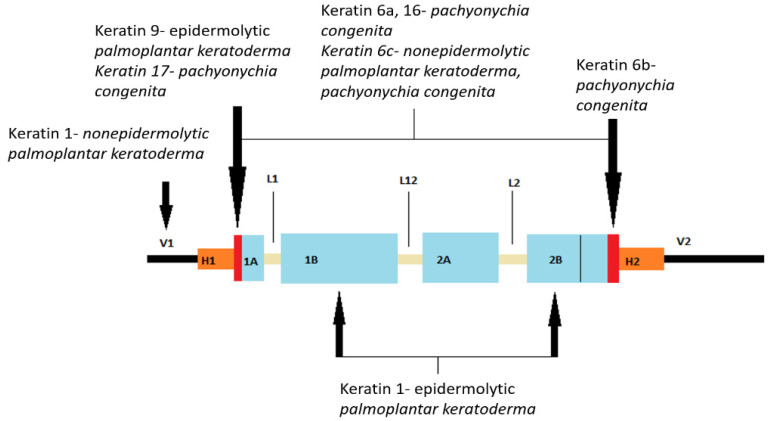
Representation of the secondary structure of keratin with marked mutation sites for some of the palmoplantar keratodermas (PPKs). The human genome encodes 54 functional genes for keratin, which are located in two groups on chromosomes 12 and 17. Keratin 1, being a fibrous protein, consists of a secondary protein structure divided into three domains: the N-terminal main domain, the central α-helical rod domain, and the C-terminal tail domain). The main domain further consists of three subdomains: end subdomains, variable subdomains (V1), and homologous subdomains (H1). The central rod domain consists of four α-helical subdomains (1A, 1B, 2A, 2B) connected by β-rotation regions (L). The N-terminal part of subdomain 1A is called the helix initiation motif initiating motives and the C-terminal part of subdomain 2B the protein coil termination motif. (V1 and V2, variable domains of protein head and tail; H1 and H2, homologous subdomains present only in type II keratins; parts marked red, initial and terminal motifs of the coil protein; 1A, 1B, 2A, 2B, α-helix coil domains; L1, L12, L2, connecting non-helical segments). (Modified according to: Arin MJ, Roop DR, Koch PJ, Koster MI. Biology of Keratinocytes. Bologna Dermatology. Pages: 876–887) [42]. “Reprinted/adapted with permission from Ref. [42]. Copyright year/2023, copyright owner’s name”/Bologna Dermatology Elsevier.

Mutations in the keratin gene cause many types of inherited palmoplantar keratodermas (PPKs), which are described in more detail later. Figure 1 shows the location of mutations in the keratin gene.

## 5. The Role of Ca^2+^ Gradient and pH in the Keratinization Process

It is important to mention the role of the Ca^2+^ gradient and pH in the keratinization process. Across the epidermal layers, there is a gradient of increasing intracellular Ca^2+^ concentration, with the peak concentration reached in the stratum granulosum. Intracellular Ca^2+^ is stored within the ER and Golgi apparatus [52]. It is worth noting that the extracellular space in the epidermis is much narrower than previously thought, resulting in the localization of most Ca^2+^ in intracellular stores [52]. The high concentration of calcium ions in these compartments is maintained by calcium pumps or channels, including SERCA2 (sarcoplasmic/endoplasmic reticulum Ca^2+^-ATPase). Therefore, mutated SERCA2 proteins can affect the concentration of calcium ions in the endoplasmic reticulum (ER) and the dynamics of Ca^2+^ in cells, which may eventually result in aberrant keratinization/epidermopoesis [53]. Furthermore, this high intracellular concentration of Ca^2+^ drops dramatically in the stratum corneum. This implies that there are specific mechanisms that release Ca^2+^ extracellularly in the viable epidermis while preventing it from diffusing into the stratum corneum. Possible mechanisms include the lipid barrier of the stratum corneum and tight junctions in the stratum granulosum [54,55].

Calcium regulates the transcription of all genes encoding proteins specific for keratinocyte differentiation. Additionally, calcium is essential in the posttranslational processing of profilaggrin to filaggrin. During terminal differentiation, several precursor proteins, including involucrin, loricrin, small proline-rich proteins, filaggrin, and keratin, are covalently cross-linked into the keratinized envelope by transglutaminase 1 in a calcium-dependent manner. Protein kinase C (PKC), which is activated by intracellular calcium concentration increase, induces markers of differentiation in granulated keratinocytes, including loricrin, filaggrin, and transglutaminase. It also decreases regulation of keratin 1 and 10 expression and specifically acts on the transition from stratum spinosum to granulosum [42]. Also, PKC alpha and delta are activated by calcium ions in both human and murine epidermis, thereby regulating the extracellular calcium-induced transcription of these differentiated genes [56].

Epidermal calcium gradient is necessary for proper epidermal differentiation and barrier formation. Calcium plays a key role in controlling the transcription of genes responsible for keratinocyte differentiation by regulating the transcription of all genes encoding proteins specific for keratinocyte differentiation. The transcription factors of activator protein-1 (AP-1) are found within numerous genes specific to keratinocytes, such as transglutaminase, loricrin, involucrin, profilaggrin, and other keratins. They play a role in regulating the transcription of various markers associated with differentiation [57]. The calcium levels needed for stage specific expression of differentiation-related proteins vary among the layers of the epidermis. Specifically, the extracellular calcium content necessary for the expression of profilaggrin, a late differentiation marker, is higher compared to the levels needed for the expression of keratin 1 and keratin 10 [58]. Apart from extracellular calcium ions, proteins sensitive to calcium are recognized for their role in promoting keratinocyte differentiation.

## 6. The Role and Expression of Intercellular Junctions and Channels in Epidermal Structures and Functions

The impact of desmosome components on the properties of keratinocytes and epidermal structure has recently been intensively investigated. Desmosomes are key intercellular compounds in the follicular and interfollicular epidermis. The specific composition of desmosomes is thought to influence their adhesive properties, and, in addition to having the ability to stabilize tissues, they also serve as signal transporters. Desmosomes contain two types of proteins—transmembrane proteins (desmogleins and desmocolins) and binding plaque proteins (plakophilins and plakoglobins), which are connected by keratin intermediate cytoskeletal filaments via desmoplakin [11]. The components of the desmosome in humans are encoded by multiple genes; there are four genes for desmoglein, three for desmocholine, and three for plakophilin. Based on the research data on the impact of desmosome components on keratinocyte properties, it was discovered that plakoglobin can, for example, form complexes with transcription factors, thereby controlling gene expression within the nucleus. Furthermore, plakoglobin may affect other cell properties, such as proliferation, migration, and apoptosis. Plakophilins can also influence cytoplasmic signaling pathways in terms of nuclear function. Tissues exposed to a significant amount of mechanical stress, such as the skin and its appendages, mucous membranes, and the heart, are often affected by desmosomal damage [42].

Adherent intercellular compounds consist of classical cadherins (E- and P-cadherins) and cytoplasmic protein complexes (α-catenin, β-catenin, plakoglobin) that bind transmembrane proteins to the actin microfilament of the cytoskeleton. It is important to note that plakoglobin can bind to both desmosomal and classical cadherins. As previously mentioned, β-catenin has a similar ability to plakoglobin and can transmit signals to the nucleus as a downstream effector of the classical Wnt pathway [42].

Tight junctions (“zonule occludentes”) are considered to “seal” the intercellular space, thus preventing the free diffusion of macromolecules, making them crucial for maintaining the barrier of the two compartments in the stratum granulosum [42].

Gap junctions are specialized intercellular connections that allow various molecules, ions (especially important for Ca^2+^ traffic), and electrical impulses to pass directly from cell to cell. One channel consists of two connectors (or chemicals), which connect in the intercellular space. Oligomerization of six connexins results in a chemical channel connexon [42].

**Figure 2:**
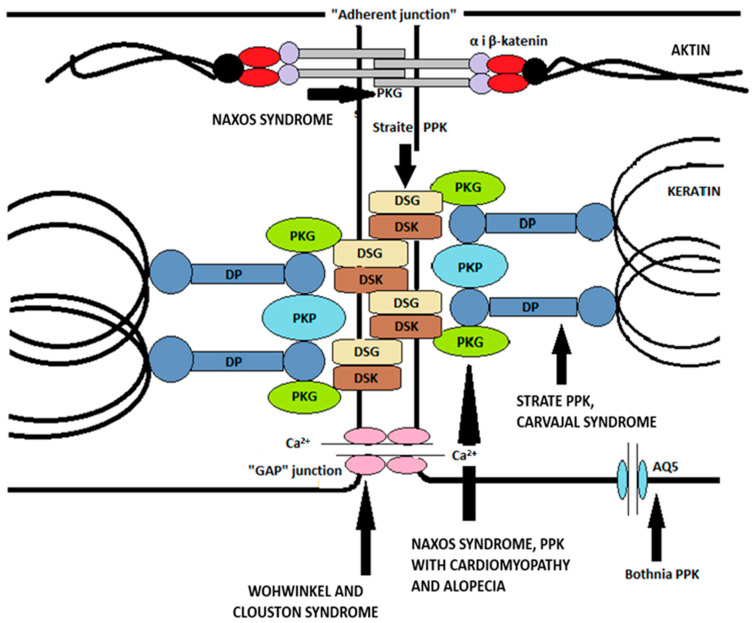
Transmembrane glycoproteins (desmogleins and desmocolins) and intermediate filaments are connected by a complex of plakoglobin, plakophilin, and desmoplakin. Other intercellular compounds are also shown—permeable, adherent junctions, and aquaporins. In addition to the listed proteins, palmoplantar keratodermas caused by mutations in the corresponding genes encoding the proteins are shown. Following the example of Arin MJ, Roop DR, Koch PJ, Koster MI. Biology of Keratinocytes. Bologna Dermatology. Pages: 876–887 [42]. Abbreviations: PKG, plakoglobin; DP, desmoplakin; PKP, plakophilin; DSG, desmoglein; DSK, desmocholine; AQ5, aquaporin 5.

The dysregulated differentiation process of keratinocytes during continuous epidermal renewal may cause various skin conditions or disorders. In this context, in skin physiology and skin diseases, aquaporins (AQs), which are a family of membrane proteins that form channels enabling the passage of water and small neutral solutes, are very important [8]. Some AQs can, additionally, transport small solutes such as glycerol or urea (e.g., AQ3, 7, 9, and 10), while others are selectively permeable to water (e.g., AQ1, 2, 4, 5, and 8) [59]. The water content in the skin is extremely high in the basal layers of the epidermis (∼75% water) and decreases sharply in the stratum corneum, which contains only 10–15% water [60]. Corneocytes, as “dead” cells, are thought to have less need for intracellular water supplies compared to basal layer cells, for which a high water content in the cytoplasm is crucial for differentiation and physiological function. The discontinuity in water content observed between the stratum granulosum and the stratum corneum is essential for maintaining the structure of the epidermis. Corneocytes form a barrier that regulates water permeability, with the hydrophobic lipid extracellular layer being crucial in human skin. AQ3 is abundantly expressed in keratinocytes in the stratum basale and spinosum but partially disappears in the stratum granulosum and is completely absent in the stratum corneum. This is consistent with recent knowledge about the role of AQ3 in keratinocyte proliferation [59]. In contrast, AQ5 shows strong localization on the plasma membrane of keratinocytes in the stratum granulosum and sweat glands [61]. According to recent research, overexpression of AQ5 in keratinocytes could promote keratinocyte proliferation and dedifferentiation but not affect apoptosis, suggesting that AQ5 mediates a balance between epidermal keratinocyte proliferation and differentiation and may play an important role in maintaining keratinocytes [62]. It has also been shown that the keratin genes 5 and 4, expressed in the stratum basale, are upregulated by AQ5 overexpression. In addition, AQ5 has also been shown to play a role in improving the organization and stability of intracellular microtubules, which is essential for the skin of the palmoplantar region, given its requirements to withstand higher levels of mechanical stress and the need for better adhesion and more robust structure [63,64]. Thus, Figure 2. illustrates the main intercellular compounds mentioned earlier and their associations with specific types of palmoplantar keratodermas caused by mutations of the individual compounds.

## 7. Genetic Basis of the Keratinization Process

The genetic code for the keratinization process is located on chromosome 1. So, genes required for the formation of terminally differentiated epidermis are located on chromosome 1q21 in the epidermal differentiation complex. The transcriptional regulation of this complex is under strict control and is one of the main targets of the differentiation cascade. Therefore, the structural changes that occur in the keratinization process are regulated by signaling pathways, transcriptional and epigenetic factors, and post-transcriptional regulation [14].

The p63 protein, encoded by the gene of the same name, has at least six different isoforms that activate or suppress transcription [42]. One of the transcription factors encoded by this gene, Zinc Finger Protein 750 (ZNF750), is crucial for the expression of the second transcription factor, Krüppel-like factor 4 (Klf4), which plays a major role in gene expression for granular layer bodies [65]. In addition, P63 also induces the expression of epidermal keratins 5 and 14 [42]. Another key function of p63 is to suppress the expression of cell cycle inhibitors and mediate exit from the cell cycle, which promotes basal layer keratinocyte proliferation. P63 also synergizes with Notch signaling to induce keratin expression 1.

“Grainyhead-like” transcription factors are also promoters of keratinocyte differentiation and are required for efficient keratinous envelope formation, in part by regulating transglutaminase-1 activity [23].

Furthermore, protein-1 (AP-1) transcription factors are present in many keratinocyte-specific genes, including transglutaminase, loricrin, involucrin, profilaggrin, and keratin 5, 6, and 14 genes, and regulate the transcription of various differentiation markers that may be associated with its increased expression in the nucleus of keratinocytes in the stratum granulosum [66].

Epigenetic processes also regulate the process of keratinization. Methylated DNA is usually associated with repressive transcriptional events, which is important in inhibiting the premature expression of differentiation genes in the stratum basale [14].

## 8. Skin Conditions/Disorders and Related Histological Findings Associated with the Altered Process of Keratinization

Disorders and disruptions of the keratinization process and subsequent desquamation are responsible for various dermatological conditions, thus comprehending the process of keratinization is extremely important in understanding the pathogenesis of these conditions. Keratinization disorders may impact both the skin and the mucous membranes, and their clinical pictures vary significantly depending on the etiology and the extent of keratin distribution [5,14]. The histological features associated with keratinization disorders are predominantly dyskeratosis, parakeratosis, and hyperkeratosis (Table 1) [44]. Dyskeratosis refers to the abnormal, premature keratinization occurring within cells or cell groups below the stratum granulosum. Parakeratosis stems from a keratinization process that is both accelerated and incomplete, resulting in the retention of residual nuclei within the stratum corneum. In parakeratosis, the turnover time is reduced to 3–4 days. Involucrin, a marker of terminal differentiation, appears in stratum spinosum. Hyperkeratosis refers to the excessive accumulation of keratin and thickening of the cornified layer or disorders related to the proper detachment of the stratum corneum [67].

In some cases, genetic defects in proteins like filaggrin, keratin, and keratohyalin, as well as enzymes responsible for cell cohesion in stratum corneum, along with molecules with roles in the signaling pathway in stratum spinosum, may underlie this pathological condition [44]. Improper desquamation and epidermopoiesis/keratinization, whether stemming from genetic mutations of factors involved in the process or arising from immune-related pathological processes, can lead to retention hyperkeratosis with regular epidermal proliferation. This differs from conditions like psoriasis, where hyperkeratosis results from increased basal layer proliferation driven by an underlying immune-related pathology [14]. Hyperkeratosis and its differential diagnoses encompass a wide range of conditions, including palmoplantar keratodermas, ichthyoses, psoriasis, atopic dermatitis, calluses and corns, seborrheic keratosis, nonmelanoma skin cancers, and others [67,68,69,70,71,72,73,74,75].

Genetic mutations and irregularities that lead to damage in components of keratinization/epidermopoiesis can be divided into the following groups: keratin abnormalities, disorders of keratinized envelope formation, disorders of gap junctions of keratinocytes (connexins), disorders of intercellular cohesion proteins (desmoplakins, desmogleins, plakophilins), and disorders of transmembrane signaling (cathepsin C) [14]. The transformation of keratinocytes from mitotically active basal cells into fully differentiated, enucleated corneocytes (in the stratum corneum) involves the expression of specific proteins and the creation of a Ca^2+^ and pH gradient at precise locations within the epidermis [63].

Furthermore, skin keratinization disorders can also be categorized in the following way: defects in the α-helical rod pattern, defects outside the α-helical rod domain, and disorders of keratin-associated proteins [5].

Defects in the α-helical rod pattern include autosomal dominant conditions, namely epidermolysis bullosa simplex (mutations in the keratin 5 and keratin 14 genes), epidermolytic ichthyosis (mutations in the keratin 1 and keratin 10 genes), pachyonychia congenita (type 1 involves keratin 6a and 16 mutations; type 2 involves keratin 6b and 17 mutations), and epidermolytic palmoplantar keratoderma (mutations in keratin 9) [51,76,77].

Defects outside the α-helical rod domain include epidermolysis bullosa with mottled pigmentation (EBS-MP) and non-epidermolytic palmoplantar keratoderma (NEPPK) [5]. So, EBS-MP results from a mutation in the non-helical head domain of keratin 5 and typically presents with acral, non-scarring blisters, along with hyper- and hypopigmented macules [78]. Also, NEPPK is a rare disorder that results from a genetic mutation in the N-terminal of keratin 1, a region that binds to desmoplakin, and clinically presents with keratosis of the palms and soles [5].

Disorders of keratin-associated proteins is a group that encompasses more prevalent conditions, including ichthyosis vulgaris, lamellar ichthyosis, pemphigus, psoriasis, and Darier disease [5,70,78,79,80]. Diseases from the group “ichthyoses” share the feature of dry, rough skin with scaling. Ichthyosis vulgaris results from an autosomal dominant mutation in the filaggrin gene, and lamellar ichthyosis results from mutations in transglutaminase-1, the major cross-linking enzyme in the stratum corneum [76,79]. Conversely, pemphigus diseases include autoimmune-mediated disorders that, by targeting cell-adhesion molecules of the cadherin family, cause blistering in the mucocutaneous membranes [71,81].

Psoriasis, a highly prevalent condition driven by immunological and genetic factors, manifests with hyperkeratotic, erythematous plaques covered by silvery scales, which are commonly distributed on the scalp, elbows, and knees [70,80]. Darier’s disease develops due to a mutation in the gene encoding the intracellular calcium pump (ATP2A2) and is inherited in an autosomal dominant fashion. Clinically, Darier’s disease is characterized by hyperkeratotic greasy papules appearing on the skin of the trunk and the scalp [82]. Additionally, the buildup of keratin in Darier’s disease may cause a rough texture and an unpleasant odor caused by bacterial degradation [5]. Thus, in the literature, “Disorders of Keratinization” encompasses various diseases other than ichthyosis, characterized by an incorrect epidermal differentiation, including palmoplantar keratoderma, follicular hyperkeratosis, erythrokeratodermia, acantholytic dyskeratosis, porokeratosis, and others [4].

However, a large number of conditions and overlapping clinical pictures can make the diagnosis challenging. Therefore, a complete medical history, detailed clinical examination, and the wise use of skin biopsy are valuable for determining keratinization disorders and their differential diagnoses [67].

## 9. Conclusions

The epidermis is maintained through an incredibly complex and perfectly coordinated keratinization process, coupled with the process of desquamation. Numerous factors control keratinocyte differentiation, including EGF, TGF-α, KGF, interleukins IL-1-β and IL-6, high vitamin A levels, and changes in Ca^2+^ concentration. The backbone of the keratinocyte transformation process from mitotically active basal cells into fully differentiated, enucleated corneocytes, is the expression of specific proteins and the creation of a Ca^2+^ and pH gradient at precise locations within the epidermis. A profound understanding of the process of keratinization is important for the pathogenesis of many dermatological diseases—ichthyoses, palmoplantar keratodermas, psoriasis, pityriasis rubra pilaris, epidermolytic hyperkeratosis, and others. These conditions often result from improper desquamation and epidermopoiesis/keratinization (due to genetic mutations of factors involved in the process or due to pathological immune processes), leading to retention hyperkeratosis with regular epidermal proliferation.

## Figures and Tables

**Table 1:** The typical histological features associated with keratinization disorders.

	Features	Common Skin Diseases Characterized by This Histological Feature
Dyskeratosis	Abnormal, premature keratinization occurring within cells or cell groups bellow the stratum granulosum	Keratosis follicularis (Darier’s disease), Transient acantholytic dermatosis (Grover’s disease), Pemphigus foliaceus, Subcorneal pustular dermatosis (Sneddon—Wilkinson disease), Familial benign chronic pemphigus (Hailey-Hailey disease)
Parakeratosis	Keratinization process is both accelerated and incomplete, resulting in the retention of residual nuclei within the stratum corneum	Clear cell acanthoma, Inflammatory linear verrucous epidermal nevus (ILVEN), Actinic keratosis, Lichenoid drug eruption, Porokeratosis, Psoriasis, Lichen simplex chronicus
Hyperkeratosis	Excessive accumulation of keratin and thickening of the cornified layer or disorders related to the proper detachment of the stratum corneum	Palmoplantar keratodermas, Ichthyoses, Psoriasis, Atopic dermatitis, Calluses and corns, Seborrheic keratosis, Nonmelanoma skin cancers, Blashkoid epidermolytic hyperkeratosis, Nevus sebaceus of Jadassohn (organoid nevus), Spitz nevus, Lupus erythermatosus, Lichen planopilaris, Tinea versicolor, Angiokeratoma

## Data Availability

Main resources for this research were PubMed and PMC.
